# A New Computational Tool for the Phenomenological Analysis of Multipassage Tumor Growth Curves

**DOI:** 10.1371/journal.pone.0005358

**Published:** 2009-04-27

**Authors:** Antonio S. Gliozzi, Caterina Guiot, Pier Paolo Delsanto

**Affiliations:** 1 Department of Physics, Politecnico di Torino, Torino, Italy; 2 Department of Neuroscience, Università di Torino, Torino, Italy; Karolinska Institutet, Sweden

## Abstract

Multipassage experiments are performed by subcutaneous implantation in lab animals (usually mice) of a small number of cells from selected human lines. Tumor cells are then passaged from one mouse to another by harvesting them from a growing tumor and implanting them into other healthy animals. This procedure may be extremely useful to investigate the various mechanisms involved in the long term evolution of tumoral growth. It has been observed by several researchers that, contrary to what happens in *in vitro* experiments, there is a significant growth acceleration at each new passage. This result is explained by a new method of analysis, based on the Phenomenological Universalities approach. It is found that, by means of a simple rescaling of time, it is possible to collapse all the growth curves, corresponding to the successive passages, into a single curve, belonging to the Universality Class U2. Possible applications are proposed and the need of further experimental evidence is discussed.

## Introduction

The fitting of any given set of experimental data may be just an exercise to find, for practical purposes, a convenient analytical curve to represent the data or, at a much deeper level, it may aim to provide a model. In the latter case one should restrict one's attention to the raw data and analyse them independently of the field of application, in order to extract from them unbiasedly every bit of meaningful information. This tough requirement is well described by the old adage “If you torture well enough your data, they'll confess”, with the double-entendre that they might “confess” what you expect or would like to find, rather than the underlying reality. A totally unbiased procedure to compel the data to “confess the truth” may be found in the Phenomenological Universalities (PUN) approach, recently proposed by P.P. Delsanto and collaborators [1]–[4], which is briefly described in the Text S1.

Tumor growth data represent perhaps one of the most critical instances of such a predicament. In fact, due to the multifaceted complexity of tumor growth mechanisms and their interactions with the host tissue, it is important to try to learn as much as possible from the data about avascular and vascular growth, metastasis, invasion, etc. Since clinical data are usually restricted to very few points in time, one tries to gain additional information from models, such as Multicellular Tumor Spheroids (MTS) [5]–[8], or from *ex vivo* experiments of transplants in lab animals, such as mice [9]–[11]. Although MTS experiments boast some inherent advantages, in the present contribution we are mostly concerned with the latter, since they are likely to be a better approximation to *in vivo* tumoral growth.

**Figure 1:**
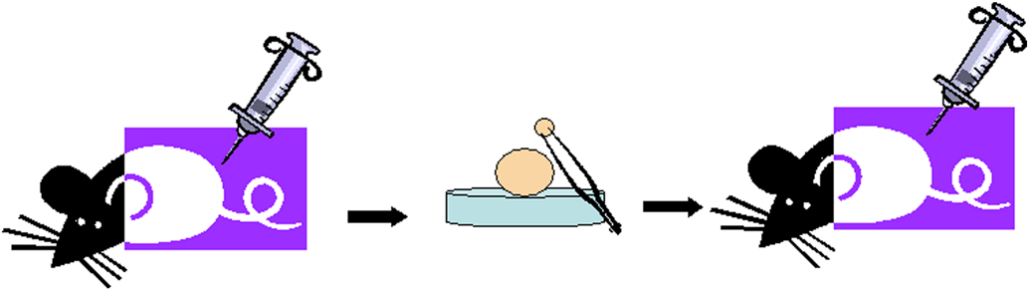
A cartoonist's view of multi-passage experiments. In MPE experiments, tumors grow following the subcutaneous implantation on the back of a lab animal (usually mice) of ∼10^6^ tumor cells (from cell cultures or from surgical resection). Tumor cells are then passaged from one mouse to another by harvesting them from a growing tumor and implanting a given number of them into another healthy animal. Once the tumor has grown above a certain volume it can be harvested again. This passage of tumor cells is repeated for multiple rounds (McCredie et al. [10] report the case of a spontaneous mammary tumor in a C3H mouse, from which the first syngenic transplant was done in 1946 and which has been serially transplanted in the C3H/HeJ strain, reaching in 1971 the 900^th^ generation!). The idea of taking a very small fraction of a spontaneous tumor mass and repeateadly transplanting it in a new host seems to reproduce the ideal situation of unlimited resources, and therefore should give us some insight about unrestricted tumor growth.

The amount of information which can be retrieved from a given dataset is obviously related to the number of experimental “points”, just as in a system of 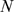 equations in order to solve for *M* unknowns it is necessary that 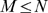. In fact, in order to reduce the effect of experimental errors, it is usually desirable that 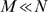. However, if new datapoints are added too close to the old ones, little information is gained, although the overall statistical accuracy may improve. For this reason multi-passage experiments (MPE) are performed, as a tool to study the long-term evolution of grafted tumor lines: see Figure 1.

## Methods

It is generally assumed that tumors originate from a “seed” and grow by cell duplication, therefore following in the first phase an exponential growth law. As long as no mechanical nor nutritional restrictions apply, they go on replicating with a constant duplication time. After a while, however, host and other constraints force the development of a necrotic core, and growth slows down towards some asymptotic level of saturation. This behaviour is well described by the well known Gompertz law [12], which has been heuristically used for more than a century in biology and other disciplines. Most aggressive tumors overcome nutrients deprivation by means of angiogenesis, and the neo-vascular network partly supports growth, as discussed by C Guiot et al. [13], following the model of G.B. West and collaborators [14]–[17]. The mechanical pressure induced by the host tissue can be circumvented by tumor invasion [18] and/or metastatic diffusion [19].

**Figure 2:**
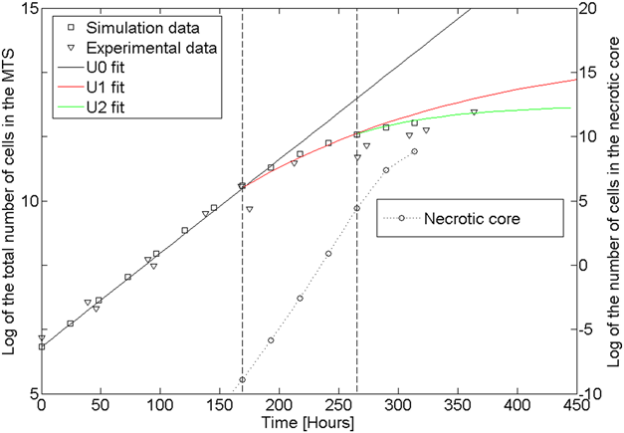
The three phases of growth of MTS's. Temporal evolution of a MTS made of EMT6/Ro mouse mammary carcinoma cells grown in a confined culture medium. The experimental data (triangles) are taken from Ref. [26]. The “squares” and “circles” correspond to the total numbers of MTS cells and necrotic cells, respectively. They have been obtained from a mesoscopic simulation [20], [27], based on the model of P. P. Delsanto and collaborators [28]–[30]. In the figure three regions may be well identified. In the first one, corresponding in the formalism of the Text S1 to the PUN class U0, there is an almost perfect exponential growth without necrotic core formation. In the second phase, which requires a better approximation as provided by U1, a bending of the growth curve towards some asymptotic level of saturation can be clearly observed: may be related to the decreasing availability of nutrients for the growing MTS. In the third phase (U2), a better agreement with the experimental data may be obtained by considering the next level of approximation (PUN class U2).

**Figure 3:**
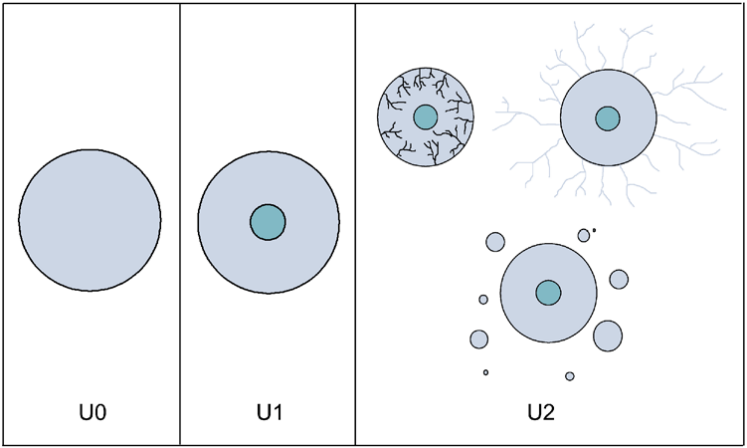
Schematic representation of the three UN phases. In U0 the MTS has no core, but starts developing it in U1. The third box represents three possible evolutionary scenarios for U2 of a tumor *in vivo* (not of a MTS), i.e. tumor invasion [18], [31], angiogenesis [32] and metastatic diffusion [19].

The progress of growth in the three phases is illustrated in Figure 2, for the case of Multicellular Tumor Spheroids [20]. The three phases are also schematically represented in Figure 3, where, however, the third phase refers to *in vivo* tumors. The three phases correspond, for the case of tumor growth, to the three PUN classes U0, U1 and U2, which are described in the Text S1. It is important to remark, however, that the progression from one class to the next one must not be understood as a “shift”, but rather as a better approximation (see Table 1 and/or Text S1, Equations 3 and 4) which becomes necessary as the tumor ages.

**Table 1:** Explicit expression of the generating functions *b(a)*, growth rate *a(t)*, and *z(t)* = ln[*y(t)*] for the three classes U0, U1, and U2.

Class	*b(t)*	*a(t)*	*z(t)*	Remarks
U0	*0*	*a_0_*	*z* = *a_0_t*	Exponential growth.
				Constant duplication time
U1	*βa*	*a_0_* exp(*βt*)	*z* = *a_0_/β* [exp(*βt*)−1]	Gompertz law.
				Growth restricted by b.c.'s and/or other constraints
U2	*βa*+*γa^2^*	*a_0_* [(1+*a_0_γ*/*β*) *e^−βt^*−*a_0_γ*/*β*]^−1^	*z* = −1/*γ* ln[1+*a_0_γ*/*β* (1−*e^βt^*)]	Generalization of the West law [14]–[17].
				Emergence of fractal properties in the solution

*y(t)* represents the given dataset, in our case the mass (or volume) of the transplanted tumor at each passage *n*.

Let us now assume that we always transplant “young” tumors, i.e. that a new seed of approximately the same mass 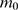 is taken each time after a short time 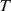 (e.g. ten days) after each transplant. Then we can assume to have always an exponential growth law with approximately the same rate *C*. It follows that, at the time 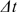 after 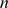 transplants, i.e. 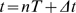 the tumor mass will be given, at least as a first approximation, by
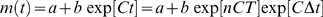
(1)


In order to have *m(t) = m_0_* at the outset of each new transplant, we must have 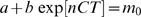 for every value of 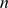, hence 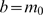 and 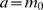 (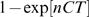). It follows:
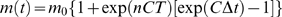
(2)where 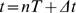 and 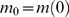. Equation (2) shows that the exponential trend is corrected by a term (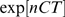), which accounts for the real age of the tumor and increases at each transplant, thus accelerating the growth. I.e., at each transplant the curves become steeper and steeper. Other explanations for the growth acceleration are also possible, e.g. a lack of accumulated toxic wastes in the new host and/or the emergence of a more aggressive subpopulations.

It is interesting to note that the Equation (2) can be viewed as a Taylor expansion of the Gompertzian law at the first order on 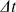.

If, after a limited number 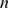 of transplants, we wish to follow the tumor growth for a large time 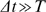, then it is no longer possible to assume that we have a purely exponential growth, since we eventually enter into phases one and two. Thus, for a best fitting of the experimental curves the formalism of class U2 must be adopted (Text S1, Equation 5). Also, in order to keep into account the growth acceleration after 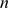 passages, as discussed before, it is convenient to renormalize the physical time 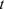, using instead
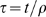
(3)where the acceleration parameter *ρ* increases with the number of transplants 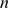.

## Results

**Figure 4:**
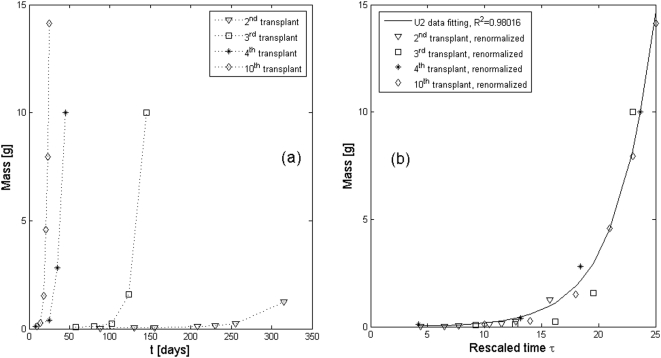
MPE results from G. G. Steel. Up to 10 transplants of cells from the tumoral line rat fibroadenoma have been performed, but the curves corresponding to only four of them have been reported [9]: see (A). As discussed in Methods, the averaged growth curves become increasingly steeper with successive passages, due to the aging of the newly transplanted tumor cells. By rescaling the time (see Equation 3) with a suitable choice of the parameter *ρ*, we obtain a plot (B), in which all the curves are collapsed into a single one, belonging to the PUN class U2. The fitting parameters are reported in Table 2.

As a first instance of application of our approach, we consider the classical work of G. G. Steel [9], who performed up to ten “passages” of cells from the tumoral line rat fibroadenoma, (see Figure 4). As discussed in the previous Section, the growth curves became increasingly steeper at each successive passage, as it can be inferred also from an analysis of the fitting parameters (see Table 2). In fact, the y-derivative in *t = 0* is steeper at each successive passage. However, contrary to what one could expect, the asymptotic value of the tumor mass, given by (see Text S1, Equation 5)
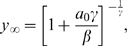
(4)decreases with the number of transplants, since *β*<0.

**Table 2:** U2 fitting parameters for the data from [9].

Data from [9]	2^nd^ transpl.	3^rd^ transpl.	4^th^ transpl.	10^th^ transpl.
*β*	−0.013	−0.019	−0.025	−0.043
*γ* [×10^−5^]	0.005	0.012	0.643	0.868
*a_0_γ/β* [×10^−3^]	−0.004	−0.005	−0.090	−0.158

For the second and third transplants the values of *γ* are negligible. They begin to be appreciable (although still very small) only in subsequent transplants. Correspondingly the value of *p* starts being different from one (thus causing a small level of fractality) only in the latter.

**Figure 5:**
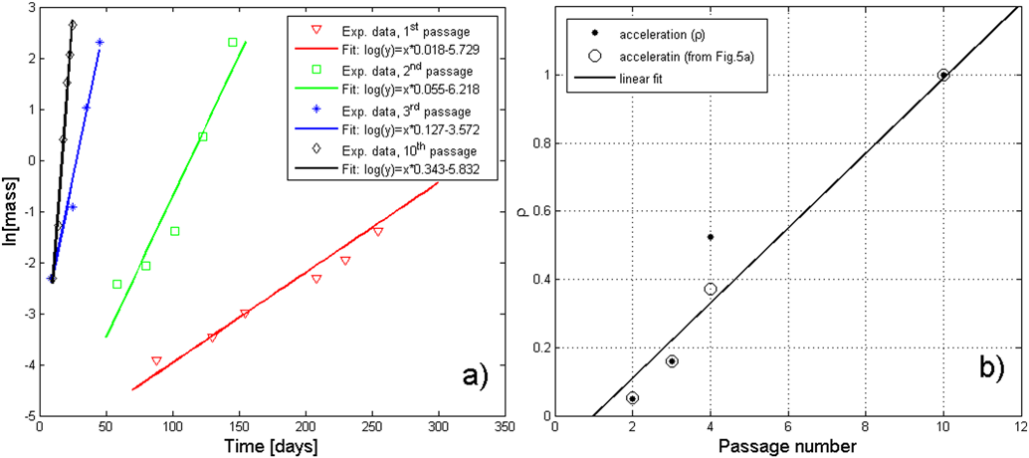
Variation of the “acceleration” parameter *ρ* with the passage number. In (A) the experimental data from [9] have been plotted in a semi-logarithmic plane and fitted, in a first approximation, with a linear function. For each passage *n* it is then possible to extract from the angular coefficients the values of the acceleration parameter *ρ*, obtained experimentally (A) and theoretically (by collapsing together the four curves of Figure 4A as done in Figure 4B).

A very interesting follow-up to Steel's data, which to our knowledge has not been recognized by other researchers, is the following. If we “renormalize” his data by means of a simple rescaling of the time (see Equation (3)), after a suitable choice of the parameter *ρ* we obtain a plot (Figure 4B), in which all the curves corresponding to the various passages, are collapsed into a single one, belonging to a very good approximation (*R^2^* = 0.98016) to the PUN class U2 (Text S1, Equation 5). This result, besides its intrinsic interest (since it means that a tumor after transplant keeps growing with the same law as before, as discussed previously), is also of great relevance for our analysis, since it allows the accumulation of a large number of independent datapoints. It may also be interesting to observe that the parameter *ρ* (which can be obtained from Equation 3 and Figure 5A) seems to vary linearly with the number of passages, as shown in Figure 5B. However, since only the data for four transplants were reported, no firm conclusion can be reached on this point.

**Figure 6:**
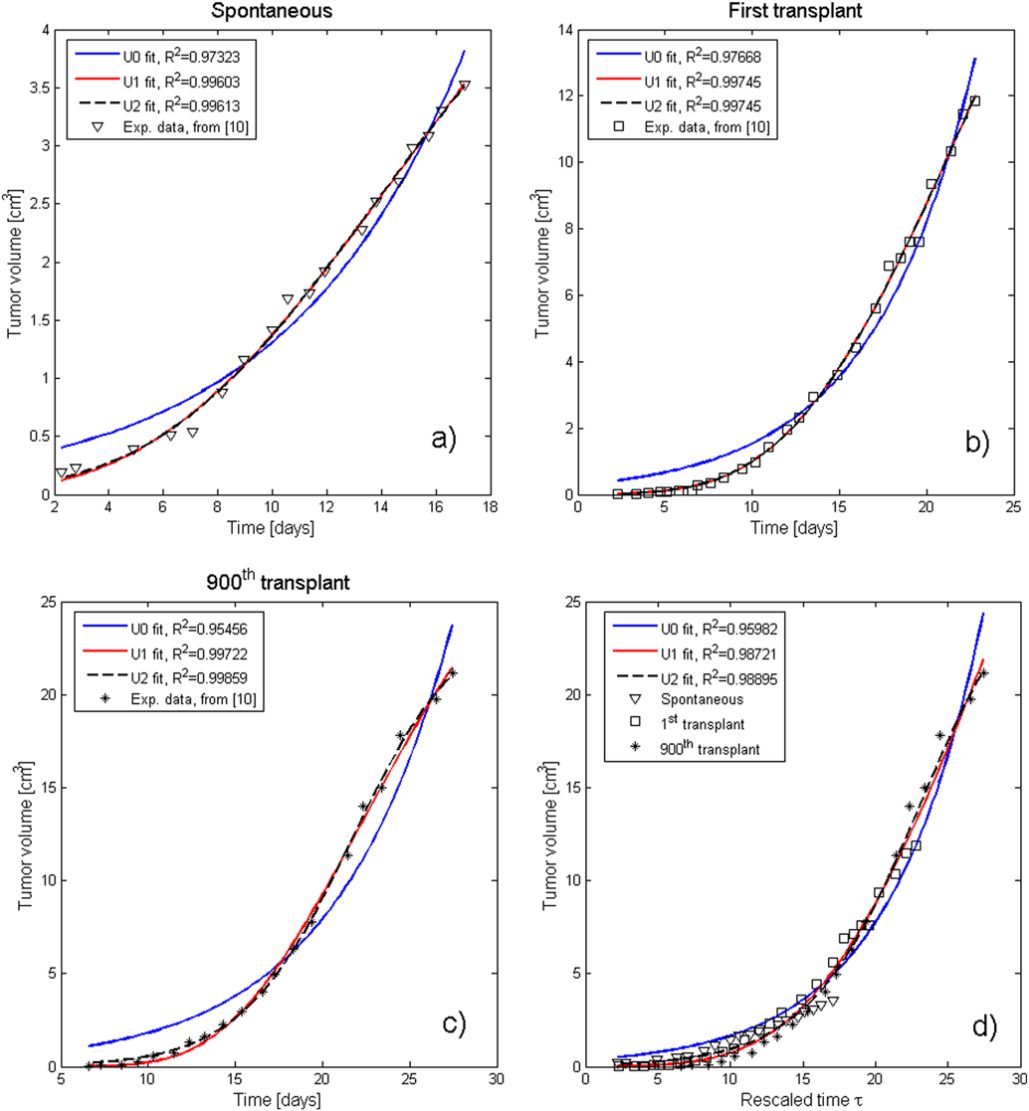
MPE results from McCredie et al. [10]. As many as 900 transplants of cells from the tumoral line C3H rat mammary carcinoma have been performed, but the curves corresponding to only the original one, the first and the last (900^th^) transplant have been reported: (A–C), respectively. For all of them the fits of the data corresponding to the classes U0, U1, and U2 have been included. There is an obvious improvement when one goes from U0 to U1 and a much smaller one from U1 to U2. The latter, in fact, yelds an almost perfect agreement with the experimental data. As in the case of Steel's data, in (D) the growth curves are normalized and displayed as a single group of data vs. the rescaled time *τ*. Here, as in Figure 4B, a U2 curve gives an excellent fit to the regrouped data. It is indeed remarkable that after as many as 900 transplants the tumor still grows with the same law (a part from the time “rescaling”). The fitting parameters are reported in Table 3.

In order to confirm the validity of our approach, we have extended it to another set of data, from the paper of McCredie et al. [10]. In their paper the rate of growth of the C3H spontaneous mammary carcinoma in the mouse is compared with that of its first and 900^th^ generation syngeneic transplants (see Figure 6). The results of our analysis are presented in Figure 6 and the fitting parameters reported in Table 3.

**Table 3:** U2 fitting parameters for the data from [10].

Data from [10]	Spontaneous	1^st^ transpl.	900^th^ transpl.
*β*	−0.030	−0.035	−0.134
*γ* [×10^−4^]	0.109	0.115	0.219
*a_0_γ/β* [×10^−3^]	−0.906	−0.812	−0.929

The “spontaneous” growth curve was recorded differently from the successive transplants (see Figure 6A compared to Figure 6B, 6C, and 6D). Hence a different value of *a_0_*. Due to the very small values of *γ*, the improvement between U1 and U2 in Figure 6 is only marginal. The normalized values of the acceleration parameter *ρ* for the three cases are 0.27, 0.36 and 1., respectively.

The MPE growth acceleration has also been observed by other researchers [21], [22] who, unfortunately, did not report the detailed growth data necessary for a computational analysis. It is important to remark that the timing of the re-implantation as well as the dimension reached by the tumor before transplantation may be very different in different experiments. Allowing the tumor to become large before transplantation invalidates the assumption of ‘unrestricted growth’ so that the tumor-host interaction becomes more and more important. This makes multipassage models interesting for understanding other biological mechanisms involved. For instance, after implantation, subpopulations of other apparently uniform cell populations may develop, with different properties of proliferation, migration, and metastasis [23].

In other development, Shen et al. [24] studied the cellular adjustment of gastric cancer for hepatic metastasis in successive orthotopic implantation models. The authors compared the parental cell line (YCC-16) with those obtained by inoculation into nude mice after 1, 2 and 3 passages (S1L1, S2L2 and S3L3 respectively). They found that, although slower than the parental line, the doubling time decreased from S1L1 to S3L3 while clonogenity increased. A progressive increase in the expression of matrix-metalloproteasis MMP-2 ( i.e. the ability of invasion) was observed. Also Beniers et al. [21], investigating five lines of renal tumors transplanted in mice after reaching al least 1 cm in diameter, were able to show differences in ‘tumor aggressivity’, i.e. clonogenity, metastasis capacity, etc., between the 5^th^ and the 15^th^ passage.

## Discussion

The accelerated growth of transplanted tumors at each successive passage challenges our understanding of tumor development. In fact, it is well known that multipassaging in *in vitro* setups does not show any remarkable variation in growth rate [23]; when the same cell line is implanted in animal models, on the contrary, the growth accelerates significantly at each new passage.

In this contribution we have analyzed two instances of multipassage experiments in mice: the classical studies of Steel [9] and of McCredie et al. [10]. As a tool for the analysis of their datasets we have applied the Phenomenological Universalities approach [1], which has been fruitfully utilized for application in different fields (among them: auxology (human growth) [3], elastodynamics [4] and fracture mechanics [25]). Perhaps the most important PUN class studied to date is the class UN, which at the first level (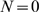), corresponds to unrestricted exponential growth. At the level 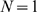, it yields the Gompertz law, which has been used, up to few years ago, to fit almost all growth phenomena. Finally, at the level 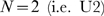 (i.e. U2), it successfully predicts the fractal properties of the solution of the growth equation at larger times. By applying the PUN approach to the data of Steel [9] and McCredie et al. [10], we have found that the class U2 describes extremely well both their datasets, although with accelerated time scales.

We have also found an explanation for this acceleration. In fact if, as in the two cases been analyzed, the transplants are always performed after a short time 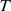, with only relatively few cells reimplanted into a new healthy and well oxigenated host, their growth occurs in virtually unrestricted conditions. From a mathematical point of view we keep having always approximately the same exponential growth 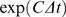 during each successive transplantation. But the cumulative effects of a certain number 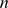 of transplants yields a term 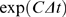, which may be responsible for the accelerated growth (see Equation 1). In other words, the tumor cells are as old as the seed taken at the very beginning and they grow correspondingly faster. This result may have important consequences in clinical practice. In fact, if one or more parameters of the system do not change upon transplantation, they could be considered as a sort of ‘fingerprints’ of a specific tumor. Then, a very important corollary to our work could perhaps be the study of metastatic diffusion, since the parameters of secondary neoplasies would be as well related to the ones of the primary tumor. Their growth rate would be speeded (as discussed for the MPE's), albeit possibly also slowed down by host and other restrictions. Unfortunately, to our knowledge, no recent data on MPE's are available. Further experimental evidence is, of course, needed, in order to confirm the validity of our conjecture and, in particular, to ascertain the dependence of the acceleration parameter *ρ* on the passage number, which in Figure 5b has been assumed to be linear on the basis of a limited number of data [9].

## Supporting Information

Text S1 The Phenomenological Universalities Approach - The Fromalism.
(0.11 MB DOC)
