# Supplementary material for: A New Computational Tool for the Phenomenological Analysis of Multipassage Tumor Growth Curves
Source: PLoS One. 2009 Apr 27;4(4):e5358. doi: 10.1371/journal.pone.0005358 (PMC2670507; doi:10.1371/journal.pone.0005358)
Supplement: Text S1 — The Phenomenological Universalities Approach - The Fromalism. (0.11 MB DOC) [file pone.0005358.s001.doc]

### TEXT S1: The Phenomenological Universalities Approach – The formalism

In addition to their epistemological interst, PUN’s can be applied as a tool for the solution of a variety of problems [1-4]. We restrict ourselves here to discuss their application to the following one: given the string of data , , representing e.g. the temporal evolution of the mass or volume of the tumor, then

1. find a best fitting function , based not on an “ad hoc” numerical procedure, but on a general formalism independent of the field of application.
2. from the fitting function obtain a model for the proposed phenomenology.

In typical nonlinear growth problems, one may assume that

, (1)

where and represents the growth rate. By defining , Eq. (1) becomes simply

(2)

In general , but in the following we restrict ourselves to the case . The cases and given by a sum of contributions from two terms, and , each depending on only one of the two variables, are discussed in [3] . We then assume that

, (3)

with

(4)

If we truncate this expansion at the *N*-th term and obtain hereby a good fitting of the dataset , then we call UN the corresponding PUN class. By solving the two differential equation , and Eq.(2), one obtains the results for and , which are summarized in Table 1 for .

From Tab. 1 and we obtain the U2 growth law

(5)

where .

It can be easily proven that satisfies the Ordinary Differential Equation

(6)

where Equation (6) can be interpreted as an energy conservation law in which the input energy is partly used for growth (up to saturation) and partly for metabolic consumption. Since is in general non integer, Equation (6) implies a fractal nature of the energy source. Other interpretations of Equation (6) are, of course, possible, depending on the field of application.
